# Detection of Morphological Abnormalities in Schizophrenia: An Important Step to Identify Associated Genetic Disorders or Etiologic Subtypes

**DOI:** 10.3390/ijms22179464

**Published:** 2021-08-31

**Authors:** Anne-Clémence Priol, Laure Denis, Gaella Boulanger, Mathieu Thépaut, Marie-Maude Geoffray, Sylvie Tordjman

**Affiliations:** 1 Pôle Hospitalo-Universitaire de Psychiatrie de l’Enfant et de l’Adolescent (PHUPEA), Centre Hospitalier Guillaume Régnier, University of Rennes 1, 35000 Rennes, France; l.denis@ch-guillaumeregnier.fr (L.D.); gaella.boulanger@gmail.com (G.B.); mathieu0thepaut@gmail.com (M.T.); 2 Department of Child and Adolescent Psychiatry, Centre Hospitalier Le Vinatier, 69500 Bron, France; marie-maude.geoffray@manchester.ac.uk; 3 CIC (Clinical Investigation Center) 1414 Inserm, Centre Hospitalier Universitaire (CHU) de Rennes, University of Rennes 1, 35033 Rennes, France; 4 Integrative Neuroscience and Cognition Center (INCC), CNRS UMR 8002, University of Paris, 75006 Paris, France

**Keywords:** schizophrenia, clinical genetics, physical examination, dysmorphic features, morphological abnormalities, minor physical anomalies, early detection, neurodevelopmental disorder, genetic disorders

## Abstract

Current research suggests that alterations in neurodevelopmental processes, involving gene X environment interactions during key stages of brain development (prenatal period and adolescence), are a major risk for schizophrenia. First, epidemiological studies supporting a genetic contribution to schizophrenia are presented in this article, including family, twin, and adoption studies. Then, an extensive literature review on genetic disorders associated with schizophrenia is reviewed. These epidemiological findings and clinical observations led researchers to conduct studies on genetic associations in schizophrenia, and more specifically on genomics (CNV: copy-number variant, and SNP: single nucleotide polymorphism). The main structural (CNV) and sequence (SNP) variants found in individuals with schizophrenia are reported here. Evidence of genetic contributions to schizophrenia and current knowledge on genetic syndromes associated with this psychiatric disorder highlight the importance of a clinical genetic examination to detect minor physical anomalies in individuals with ultra-high risk of schizophrenia. Several dysmorphic features have been described in schizophrenia, especially in early onset schizophrenia, and can be viewed as neurodevelopmental markers of vulnerability. Early detection of individuals with neurodevelopmental abnormalities is a fundamental issue to develop prevention and diagnostic strategies, therapeutic intervention and follow-up, and to ascertain better the underlying mechanisms involved in the pathophysiology of schizophrenia.

## 1. Introduction

Schizophrenia is a chronic psychiatric disorder. The diagnosis, according to ICD-10 (World Health Organization-WHO) and DSM-5 (American Psychiatric Association) criteria [1,2], is based on three main psychopathological dimensions: (1) delusional distortion of reality with positive symptomatology; (2) affective impoverishment with negative symptomatology including apathy and social withdrawal; and (3) disorganization of thought.

Schizophrenia affects 0.3 to 0.7% [1] of the world population (approximately 600,000 individuals in France) with typically adult-onset schizophrenia (AOS, at age ≥ 18 years). However, early onset schizophrenia (EOS, onset before age 18 years, prevalence of approximately 0.03% [3]) and very early onset schizophrenia (VEOS, onset before age 13 years, prevalence of approximately 0.002% [3,4]) are also observed.

Schizophrenia is viewed as a complex multifactorial disorder and is classified by the WHO in the group of the 10 diseases that cause the most disability. Current research suggests that alterations in neurodevelopmental processes, involving interactions between genetic and environmental factors during key stages of brain development (prenatal period and adolescence), are a major risk for schizophrenia [5]. Early detection of individuals with neurodevelopmental abnormalities is therefore a fundamental issue. This would make possible to identify homogeneous subgroups of patients with similar pathophysiological pathways which is a key stage for the etiological understanding of this complex disease.

Many studies have described different phases of symptoms (behavioral and adjustment problems) observed in almost half of patients with schizophrenia in the 10 years preceding the first hospitalization [6]. They are based on Larsen’s work [7] which describes three phases before the onset of the disease: (1) the pre-morbid phase in childhood or preadolescence with certain signs of vulnerability: higher level of anxiety [8], cognitive deficit, especially attention deficit [9], language delay and difficulty in social adjustment with aggressive or disruptive behavior [10]; (2) the early prodromal phase a few years before the first psychotic episode (up to 5 years before [11]) with nonspecific symptoms and perceptual abnormalities: abnormal visual perception (notably for the size of objects and their distance), change in social interactions (such as social withdrawal and isolation), verbal language impairments (such as production of maladapted words or fading), other cognitive impairments (such as feeling of thought blockage and attention deficit), and motor impairments (automatic gesture abnormalities) [11,12]; (3) and the late prodromal phase with the appearance of the first psychotic symptoms: hallucinations, disorganized speech, negative symptoms [1].

The current priority is to develop earlier diagnostic strategies to enable better clinical management and promote therapeutic interventions as soon as possible knowing that early detection and treatment of schizophrenia can improve the prognosis [13,14]. Duration of untreated psychosis (DUP) is also known to be an important prognosis factor for the quality of the long-term therapeutic response. The subsequent course of the disease is less favorable when the DUP is long [15]. The studies conducted to identify etiological factors of schizophrenia are therefore essential to allow a diagnostic orientation and appropriate early therapeutic strategies.

Neurodevelopment in humans concerns the establishment of the nervous system during embryogenesis and the following stages of ontogeny. This term includes all the processes that allow the initial development of the nervous system in utero to maturation process during childhood and adolescence. The initial hypothesis in favor of a neurodevelopmental origin of psychiatric disorders stems from the assumption that abnormalities occurring during windows of vulnerability of brain development may favor the appearance of psychiatric disorders. These windows occur at multiple times during development [16,17]. Early abnormal neurodevelopment (during embryogenesis) can lead to dysplasia of certain neuronal circuits that may be responsible for the premorbid abnormalities observed in some patients with schizophrenia [18]. Furthermore, the onset of the disease may be also linked to a later abnormal brain maturation during adolescence [19]. These abnormalities in brain development and maturation appear to be the result of interactions between genetic and environmental factors that lead to cell proliferation and migration defects, lack of synaptogenesis and myelination failure [20,21]. Patients with mutations (de novo or inherited from a parent) in candidate genes for schizophrenia would have a particular sensitivity to certain environmental factors. It may be the combination of these genetic and environmental factors that would lead to the onset of the disease. The candidate genes for schizophrenia are involved in several key stages of brain development and their function is modulated by environmental factors [22]. If prenatal neurodevelopmental anomalies are involved in the etiopathology of schizophrenia, then the symptoms that appear during adolescence should no longer be considered as the beginning of the disease but as an already evolved phase of disorder. From this postulate comes the concept of ultra-high risk (UHR) of psychosis. Individuals with ultra-high risk of schizophrenia have a genetic vulnerability to trigger the disease but symptoms of schizophrenia may not express without exposure to certain environmental factors. From a clinical point of view, this highlights the importance to follow these individuals earlier to propose prevention strategies based on close monitoring and rapid therapeutic intervention [17,23]. To develop such strategies, one must be able to identify early vulnerability factors. Markers of early neurodevelopmental abnormalities, such as minor neurological and morphological abnormalities, may play a major role in prevention strategies and early detection.

The ‘minor’ neurological abnormalities or soft signs have been of interest because their presence may reflect high neurodevelopmental abnormalities. It is noteworthy that soft signs are significantly associated with schizophrenia and not with other psychiatric disorders. They must be distinguished from ‘major’ neurological signs that correspond to motor or sensory abnormalities that may be related to dysfunction of a specific brain area. Several studies [24,25] suggest that patients with schizophrenia have neuromotor signs called ‘minor’ neurological signs—such as coordination disorders, fine and gross motor skills, and sensory integration disorders—even during the first episode. They are also present in the parents of patients and are more frequent in familial schizophrenia [26], which explains why they are proposed as endophenotypic markers for schizophrenia [27]. In addition, it has recently been shown that soft signs are more common in EOS, before adolescence, compared to AOS. EOS individuals have also significantly more soft signs than the general population, particularly motor coordination disorders and laterality disorders [28]. These signs could differentiate UHR individuals from typically developing individuals with an accuracy of 85.9% [29]. These results suggest that soft signs could be viewed as markers of a more prominent neurodevelopmental form [30]. Regarding these findings concerning soft signs, it is hypothesized that minor morphological abnormalities would also be the markers for early identification of UHR individuals. Minor morphological abnormalities in schizophrenia would reflect underlying genetic factors and would represent objective, early, and stable endophenotypic markers similarly as for soft signs [31,32,33].

The assumption that schizophrenia has neurodevelopmental origins requires studies to identify genes involved in neurodevelopmental processes as vulnerability genes for schizophrenia. These genetic studies need to direct research towards certain genetic syndromes already known and for which the risk of developing schizophrenia is higher than in the general population.

## 2. Epidemiological Studies Supporting a Genetic Contribution to Schizophrenia

Individuals with a parent with schizophrenia have a higher probability of developing the disease. Sibling studies have shown that siblings of individuals with schizophrenia have 6% risk of developing schizophrenia, 15% if they are dizygotic twins, and 50% if they are monozygotic twins. The results of the studies on adopted children showed that 6% of biological siblings adopted in a different family from their index case siblings develop schizophrenia. Heritability is estimated between 79% [34] and 82–85% [35], based on a non-Mendelian transmission profile.

### 2.1. Family Studies

Several epidemiological studies reveal a family aggregation of cases of schizophrenia, which means that the closer a related individual is to a patient, the more likely this individual is to develop schizophrenia over his or her lifespan, but the mode of transmission does not correspond to a Mendelian monogenic transmission profile [36]. Gottesman and Shields [37] have shown that, compared to the current risk of 1% in the general population, the risk of schizophrenia is about ten times higher in the first-degree relatives of a patient with schizophrenia. This risk is higher among siblings (10.1%) or children (12.8%) than among parents (5.6%). These differences could be explained by the low rate of reproduction and marriage of patients with schizophrenia [38]. The risk of developing schizophrenia for a child of two parents with schizophrenia is estimated at 46% [39]. Among second-level relatives (uncles and aunts, nephews and nieces, and grandchildren), the risk of recurrence of schizophrenia is about 3% and less than 2% for third-degree relatives. Maier et al. [40] found a risk of recurrence of 5% for first-degree relatives, 3.1% for second-degree relatives and 1.5% for third-degree relatives, compared with a risk of 0.8% for controls. The risk of recurrence of the disease appears as a function of the genetic burden, reflected by the number of subjects affected in the same family (almost 17% of recurrence, if a brother and a parent are both affected), the proximity of the relationship with the proponent and also the severity of the proponent’s clinical signs (about 21% recurrence in children of a hebephrenic patient and about 10% in children of a paranoid patient) [41].

Close relatives of individuals with schizophrenia have also a high risk of developing personality disorders (paranoid, schizotypal) or psychiatric disorders (schizophrenia, schizoaffective disorders, schizophreniform disorder, brief psychotic disorder, unspecified psychotic disorder, unipolar depression), questioning the existence of genetic factors (such as single nucleotide polymorphisms in core circadian clock genes) shared by these psychiatric disorders [42]. In addition, there are also some clinical features of schizophrenia, such as age at onset, that may be influenced by family history. Gorwood et al. [43] conducted studies in the French Reunion Island on individuals with schizophrenia (N = 663) divided according to the presence or absence of another individual with schizophrenia among the parents of the first and second degrees. The median age at onset differed according to sex and gender: men were younger (mean age = 27.8 years) than women (31.5 years). A comparison of age at onset based on family history revealed that the onset was later for women with no family history than for the other three groups (men with or without family history and women with family history). No difference appeared in the comparison of early ages for men and women with family history. Svensson et al. [44] showed that family aggregation of schizophrenia was reduced by higher age at onset. The authors created a population cohort in Sweden with 5,075,998 siblings born between 1932 and 1990. Of this cohort 16,346 cases of schizophrenia were identified. Family aggregation was measured as the risk of sibling recurrence, defined as the risk of schizophrenia in biological siblings of patients with schizophrenia compared to risk among siblings of unaffected subjects. The authors found a significant lower recurrence risk ratio for siblings of later onset cases (7.2, 95%, CI: 6.7–7.9) compared to early onset cases (10.8, 95%, CI: 9.4–12.2). Kallmann and Roth [45] compared the family history of cases of preadolescents with schizophrenia (52 twins and 50 singles younger than 15 years) with those of a comparable adult sample (691 twins index cases) and observed no significant differences between groups regarding the twin concordance rates or the schizophrenia rates for the parents (12.5% and 9.2%) and siblings (12.2% and 14.3%) of index cases. Fathers and mothers contribute equally to the rate of parental schizophrenia, whereas mono and dizygotic twins of schizophrenic index cases differ as much in concordance for schizophrenia in preadolescents (17.1% and 70.6%) as for schizophrenia in adults (14.7% and 85.8%). For the authors, these results indicate an early effect in childhood schizophrenia of the same genotype supposed to be responsible for the underlying symptoms of schizophrenia in adults [45]. The authors found a trend towards increasing the number of early cases of schizophrenia among twins and brothers of early index cases.

Finally, it is noteworthy that family studies do not allow the effects of genetic vulnerability factors to be differentiated from the effects of environmental factors, strengthening the need for twin and adoption studies.

### 2.2. Twin Studies

Cardno and Gottesman [46] found, based on several studies, that within monozygotic twin pairs (monozygotic twins share an identical genotype), the concordance rate for schizophrenia was ranged from 41% to 65% and from 0% to 28% in dizygotic twins (dizygotic twins have a different genotype). Heritability has been estimated from 80% to 85%. In their 2003 meta-analysis, Sullivan et al. [47] found heritability at 81% (95%, CI: 73–90%). Despite the heterogeneity of the results, the concordance rate appears always higher in the monozygotic twins than in the dizygotic twins (see Table 1, [39,48,49,50,51,52,53,54,55,56,57,58,59,60]). This observation should be taken with caution because this superiority of the concordance rate in the monozygotic twins may be not only related to shared genetic factors but also possibly to shared intra-uterine disturbances, more frequent in the monozygotic twins. The fact that the concordance rate in monozygotic twins does not reach 100% suggests that environmental factors may modulate the effects of genetic factors.

Comparing offspring of discordant monozygotic twins for schizophrenia, Kringlen and Cramer [61] have shown that the risk of schizophrenic disorders in the offspring of affected monozygotic twins is not significantly different from the risk in offspring of non-affected monozygotic twins. According to Gottesman and Bertelsen [62], the risk for schizophrenia among the descendants of monozygotic twins is very similar in the 14 children of schizophrenic identical twins (16.8%) and in the 24 children born from non-schizophrenic twins (17.4%). These results suggest the existence of epigenetic and/or environmental factors that are not shared.

### 2.3. Adoption Studies

Adoption studies help to separate the influence of genetic factors from environmental factors, such as studies of separately raised twins [63]. Slater and Cowie [64] found concordance of 77.6% for monozygotic twins in separated lives and of 91.5% for those raised together. In adoption studies the prevalence of schizophrenia was found to be higher in adopted children with affected biological relatives than in adopted children without affected biological relatives, both living in the same environment. From a sample of 5483 subjects adopted early in life by unrelated people, Kety et al. [65] compared the biological and adoptive relatives of 33 of these individuals who became schizophrenic to 33 non-schizophrenic adoptees. They found that 9% of the biological relatives of individuals with schizophrenia had schizophrenia spectrum disorders compared with 2% of the biological relatives of control subjects. Lichtenstein et al. [66] used Swedish registers over several generations to study the risks of schizophrenia, bipolar disorder and their comorbidity in biological and adoptive parents, descendants, siblings, and half-siblings of patients. First-degree relatives with schizophrenia or bipolar disorder were at increased risk for these disorders. Half-siblings had a considerably increased risk, but significantly less than siblings. In the analysis of parents of individuals with bipolar disorder, there was an increased risk of schizophrenia for all relationships, including adopted children of biological parents with bipolar disorder. The authors conclude that schizophrenia and bipolar disorder share common genetic factors. Higgins [67] compared two groups of children born from mothers with schizophrenia, one raised by their biological mother and the other one by an adoptive mother; they found that the risk of schizophrenia was the same in both groups. These results suggest that the risk for schizophrenia depends more on genetic factors than on environmental factors.

## 3. Genetic Disorders Associated with Schizophrenia

An extensive literature review was conducted to identify genetic disorders most frequently associated with schizophrenia. The results are presented in Table 2 and differentiate genetic disorders of chromosomal origin from those of monogenic origin. This table includes only genetic syndromes involving a monogenic disorder or chromosomal rearrangement, and thus does not include polygenic causes and genetic disorders related to epigenetic mechanisms. The results are not exhaustive.

The estimated rate of genetic disorders in the schizophrenia population and the estimated rate of schizophrenia in patients with the genetic mutation are provided when the data are available. To examine the neurodevelopmental hypothesis, the age at onset of schizophrenia (VEOS, EOS, AOS) was also indicated. The other possible associated psychiatric and psychological disorders as well as the morphological abnormalities characterizing the genetic syndrome are detailed.

## 4. Genomics and Schizophrenia: Structural (CNV: Copy Number Variant) and Sequence (SNP: Single Nucleotide Polymorphism) Variants

As we have seen, epidemiological data on twins and adopted children support a significant role of genetic factors and the involvement of environmental events in the development of the disease, suggesting that schizophrenia is a neurodevelopmental disorder. These findings led researchers to conduct many studies on genetic associations to identify risk factors for schizophrenia.

Very early-onset schizophrenia (VEOS) is rare (prevalence of approximately 0.002% [3,4]), more severe (especially when VEOS is associated with catatonia [101]), and more frequently associated with genetic factors than schizophrenia with later onset [102]. In 67% of children with childhood-onset schizophrenia, premorbid motor as well as social and language impairments were observed [103]. Individuals with a very high genetic risk for schizophrenia are more frequently carriers of chromosomal rearrangements. Among these rearrangements, the 22q11.2 deletion and (1:11) (q43, q21) translocation are the most described in cytogenetic studies. The 22q11.2 deletion concerns 1.5 to 3 Mb—i.e., 30 to 40 genes [104]—particularly the *COMT* (Catechol-O-Methyltransferase) gene involved in the degradation of neurotransmitters including dopamine [105]. This region is associated with velocardiofacial syndrome (VCF), also known as DiGeorge syndrome, in which up to 30% of adults develop a psychotic spectrum disorder of schizophrenia. The translocation between chromosome 1q and 11q affects the *DISC* (Disrupted in Schizophrenia) gene on chromosome 1, which encodes a protein involved in neuronal migration and axonal growth. This genetic abnormality is associated with a risk of developing schizophrenia [91].

Subsequently, many genetic studies have been developed to identify vulnerability genes for schizophrenia. The first strategies were based on the search for candidate genes in relation to the proposed hypotheses related to a neurobiological (dopaminergic, serotoninergic, glutamatergic) or neurodevelopmental disorder [106]. The hypothesis of schizophrenia as a neurobiological disorder has focused since the 1960s on presynaptic dopaminergic dysfunction, and more precisely since the therapeutic effects of the first antipsychotics were mainly observed for positive symptoms of schizophrenia due to their antagonistic action on dopamine D2 receptors. Today, the dopaminergic neurobiological model develops around two excitation/inhibition imbalances in the cortical circuits: hyperactivity of the mesolimbic dopaminergic neurons (sub-cortical) underlying the positive symptoms (delusional ideas), and hypoactivity of the neurons mesocortical dopaminergic (prefrontal cortex) underlying negative (blunted affect) and cognitive symptoms [107]. However, the efficacy of antipsychotics in the control of deficit symptoms of schizophrenia (negative, depressive, and cognitive symptoms) is negligible, and one-third of patients do not respond to antipsychotic treatments [108], suggesting that schizophrenia is not exclusively due to a primary dysfunction of the dopaminergic system.

The pharmacological approach has questioned associations between schizophrenia and glutamate, which is the major excitatory neurotransmitter in the central nervous system in mammals (40% of synapses). The interest of glutamatergic transmission in schizophrenia arises initially from the observation of the effects of psychotomimetic drugs, phencyclidine (PCP) and ketamine, which are noncompetitive NMDA glutamatergic receptor antagonists. These drugs, at subanesthetic doses in healthy volunteers induce similar symptoms to the ones observed in schizophrenia [109,110,111]. Notably, ketamine, which is a dissociative anesthetic used in human and veterinary medicine since the 1970s, has the particularity of inducing symptoms close to a first psychotic episode of schizophrenia, including positive symptoms (perceptual illusions and distortions), negative signs (blunted affects and disorganization of thought), and cognitive disorders (impairments in immediate and delayed recall, verbal fluency disorder, attention deficit disorder) [112]. Glutamate can act on two major types of glutamatergic receptors: the postsynaptic iGluR ionotropic receptors, ion-permeable ion channels (Na, K Ca), or the metabotropic mGluR receptors which are G protein-coupled. The metabotropic mGluR receptors are subdivided into three groups: the group I (mGluR 1 and 5) receptors are mainly postsynaptically coupled with phospholipase C, whereas the group II (mGluR 2 and 3) and group III (mGluR 4; 7 and 8) receptors are presynaptically coupled with adenylate cyclase [112,113]. There are three types of iGluR receptors according to their pharmacological properties: NMDA (N-methyl-D-aspartate), AMPA (Amino-3-hydroxy-5-methyl- 4-isoxazole propionic acid) and kainate. AMPA and NMDA receptors are the major postsynaptic receptors. Activation of the NMDA receptor requires the binding of D-serine to GluN1 subunits and glutamate to GluN2 subunits, to induce channel opening at their center, calcium entry, and then a cascade of intracellular signals. Consequently, the expression of several genes is modified which allows an acute functional synaptic plasticity, and then a long term neural structural plasticity [114]. Excess of glutamatergic stimulation can become toxic, triggering a massive influx of calcium into neuronal cells, and lead to apoptotic cell death.

Binding assays are based on the systematic screening of genomes within the family with affected individuals and then within a case/control population, with the aim of establishing a significant association of polymorphisms with schizophrenia. Studies have identified several candidate loci on different chromosomal regions that may be associated with the risk of schizophrenia, including NOSAP1 (1q), RGS4 (1q), DNTBP1 (6p), NRG1 (8p), and G72/G30 (13q). The locus G72/G30 on chromosome 13 comprises the DAOA gene, which encodes the D-Amino Acid Oxidase Activator, a protein responsible for the oxidation of D-serine. D-serine is a mandatory co-agonist for the activation of synaptic glutamatergic NMDA receptor. Astrocytes and glial brain cells have, in addition to a role of architectural support, regulatory functions of transmission of information at the level of the synapse. Astrocytes can capture D-serine catabolized by DAOA. The NRG1 gene encoding Neuregulin-1 located on chromosome 8p is associated with a risk of schizophrenia. It is a complex gene of 25 exons with alternative promoters and splicing, allowing the production of multiple different proteins. Neuregulin-1 is involved in the formation of glutamatergic neuronal dendritic spines, neuronal migration, Schwann cell growth, and myelination [106]. The mutation in the NRG1 is expected to contribute to an increase in neuregulin-1 signaling and thus to NMDA receptor hypofunction [115]. Since NMDA receptors are involved in the excitation of GABAergic interneurons, a key regulator of excitation, NMDA receptor hypofunction may induce a decrease in interneuron excitation and thus a disinhibition of pyramidal cells [116]. The DTNBP1 gene encodes dysbindin, a dystrophin-associated protein complex (DAPC) that plays a role in cognitive function via the glutamatergic NMDA receptor [117]. KO mice for DTNBP1 have a behavioral phenotype sharing similarities with schizophrenia, including cognitive dysfunction, as well as impaired hippocampal long-term potentiation [118].

Linkage analyses have made it possible to establish genetic maps of schizophrenia markers covering the entire genome, without identifying robust targets with the necessary criteria of significance. Overall, the results of these genetic association studies have been disappointing with many contradictory results that are difficult to reproduce, partly because of insufficient sample sizes and therefore a lack of statistical power to meet the genome wide significance level (5 × 10^−8^) [119]. Technological advances in high-throughput sequencing (HTS) have provided the opportunity to analyze widely human genomes in case/control populations. These large-scale genetic association studies have identified hundreds of thousands of nucleotide polymorphisms (SNPs). SNPs are sequence variants and correspond to genomic locations that may contain allelic differences. The GWAS (Genome-Wide Association Studies) involved 113,075 control subjects and 36,989 individuals with schizophrenia, enabling the identification of 108 independent sites on the genome associated with the risk of schizophrenia with a significance threshold of 5 × 10^−8^ [119]. Among these loci, one is associated with the dopamine D2 receptor (*DRD2*) and several with glutamatergic neurotransmission—such as mGlu3 (*GRM3*), GluN2A (*GRIN2A*), SR (*SRR*), and AMPAR (*GRIA1*). Finally, some loci are associated with calcium signaling (*CACNA1C*, *CACNB2*, and *CACNA1l*) [120]. The *GRIN2A* gene found among the 108 loci codes for the glutamatergic receptor GluN2A subunit. KO mice for *GRIN2A* exhibit similar behaviors to those observed in animal models of schizophrenia [121]. Numerous data across the literature suggest hypofunctioning of the glutamatergic system via NMDA receptor hypofunction in schizophrenia [122]. The involvement of other receptors has also been explored, notably AMPA receptors, during migration of GABA interneurons or in cognitive functions (memory and attention). Similarly, metabotropic receptors of group II mGlu2/3 have been studied; they are auto-receptors activated on astrocytes upon excessive glutamate release into the synaptic cleft. This mechanism prevents neuronal excitotoxicity by maintaining a sub-toxic level of glutamate and allows glutamate to be recycled [111]. These genes have been investigated as a potential therapeutic target in schizophrenia such as the *SRR* gene which codes for Serine Racemase [123,124,125,126].

It appears clearly that the effects of each SNP or sequence variant are low (odds ratio < 1.1), explaining the need for large studies to obtain satisfactory statistical power. Newer microarray technologies have also detected structural polymorphic variants in which DNA segments of the genome are deleted or duplicated in multiple copies, called Copy Number Variants (CNVs). These variants appear to be associated more frequently with sporadic cases than cases of familial schizophrenia. In addition, they appear to have greater effects than SNPs on the risk of schizophrenia. The first genotype analysis of schizophrenia susceptibility loci analyzed 38,779 individuals with microarray to find the presence of CNV. They identified 1035 individuals with copy number variants of recurrent loci: 1q21.1, 15q11.2, 15q13.3, 16p11.2, 16p13.11, and 22q11.2. Sahoo et al. [127] concluded that the phenotypic effects of copy number variants associated with schizophrenia are pleiotropic and imply the existence of shared biologic pathways.

An analysis of 21,094 patients with schizophrenia and 20,227 control individuals found 8 CNVs with a highly significant association (odds ratio of 0.15 to 67.7), with deletions 1q21.1, 2p16.3, 3q29, 15q13.3, 16p11.2, and 22q11.2, as well as duplications 7q11.23 and 16p11.2 [128]. It is noteworthy that 2.5% of the patients and 0.9% of the controls carried one of these CNVs. Whereas most CNVs associated with schizophrenia encompass multiple genes, the deletion of chromosome 2p16.3 specifically affects the *NRXN1* gene encoding the synaptic cell adhesion molecule, neurexin-1, which is involved in the regulation of synaptic development and in synaptic transmission [129,130,131]. Concerning Sahoo et al. and Marshall et al.’s findings [127,128] of CNVs associated with schizophrenia, several authors have confirmed these results and more details are described below in this article for the following loci: 1q21.1, 3q29, 7q11.23, 22q11.2, 15q11.2, and 16p11.2. The duplication of the chromosome 1q21.1 region (approximately 1.35-Mb) was reported in individuals with schizophrenia and tetralogy of Fallot, and the reciprocal deletion was also identified [93,94,95]. The morphological abnormalities observed in this genetic disorder are detailed in Table 2. Mulle et al. [132] identified a region in chromosome 3q29 involving CNV significantly associated with schizophrenia. The microdeletion of the chromosome 3q29 region (approximately 1.5 Mb) contain 22 genes, including *PAK2* and *DLG1*, which may play a putative role in the psychiatric manifestations of the 3q29 deletion syndrome. Mulle et al. [132] reported a 1.4 megabase duplication on the chromosome 7 (7q11.23) that increases the risk for schizophrenia over 10 times. A reciprocal 7q11.23 duplication was also observed in schizophrenia by Kirov et al. [85]. It is noteworthy that the 7q11.23 deletion corresponds to the Williams–Beuren syndrome, a multisystem neurodevelopmental disorder reported to be associated with autism and including speech delay, craniofacial anomalies, and increased incidence of congenital anomalies (for a review, see Tordjman et al. [133,134,135]). Mervis et al. [136] found that patients with reciprocal 7q11.23 duplication syndrome had significantly higher levels of separation anxiety compared to patients with Williams–Beuren syndrome and to the general population, which could be of interest regarding anxiety problems reported often in patients with schizophrenia [137]. The 22q11.2 deletion syndrome appears to be a recurrent genomic disorder distinct in fact from DiGeorge syndrome (DGS) and velocardiofacial syndrome (VCFS). About 97% of patients with DGS/VCFS have a common recurrent 3-Mb deletion of the 22q11 region [138]. Vassos et al. [72] who was the first to estimate the penetrance for schizophrenia of several CNVs, found modest rates of penetrance for all of them, except for the 22q11.2 deletion in the VCFS region which had a much higher penetrance of 55%. The deleted region of chromosome 15q11.2 is on approximately 500 kb of the Prader-Willi/Angelman syndrome critical region [79]. This region includes four genes, *TUBGCP5*, *NIPA1*, *NIPA2*, and *CYFIP1*. The deletion of chromosome 15q11.2 may contribute to the susceptibility to neuropsychiatric or neurodevelopmental problems. Von der Lippe et al. [139] and Stefansson et al. [140] found that the 15q11.2 deletion affects brain structures with similar alterations to the ones observed during the first-episode psychosis in schizophrenia. The microdeletions and microduplications of chromosome 16p11.2 region confer susceptibility to certain psychiatric disorders such as schizophrenia and autism spectrum disorder [20,141,142].

In addition to common variants with a weak effect, pharmacogenomic data have revealed rare variants (0.1 to 1%), taking the form of CNVs or SNPs, as potential candidate loci [120]. This is most often de novo mutation, responsible for a loss of function (LoF). Thus, 40 rare variants identified in the exon region coding for the NMDA receptor and in the *DRIN2C* or *GRIN2D* genes are LoF mutations. The rare or de novo CNVs appear in populations of patients with schizophrenia at a frequency twice as high as the control individuals (5.1% vs. 2.2% in controls) [143]. Of these CNVs, 4 loci were known to be associated with schizophrenia (3q29, 15q11.2, 15q13.3, and 16p11.2). Some rare CNVs are enriched in genes encoding glutamatergic proteins [144], supporting the hypothesis that rare variants responsible for loss of function in the glutamatergic pathway are important for synaptic plasticity and cognition, playing a significant role in the pathogenesis of schizophrenia [120,144].

Recently, a study combining GWAS data and data from a meta-analysis in 11,260 cases of schizophrenia and 24,542 control cases identified 50 new loci and 33 candidate genes for schizophrenia. The authors show also that genes subjected to high selective pressures are strongly enriched with common variants (SNPs), suggesting mutant-intolerant genes [145]. Mutant-intolerant genes are enriched with common variants at risk for neurodevelopmental neurological disorders such as autism spectrum disorder or intellectual disability, or other pathologies such as epilepsy, congenital heart disease, microcephaly, and obesity [120,146]. These genes account for a 30% proportion of heritability based on SNPs in schizophrenia. In addition, this study revealed five sets of independent genes associated with schizophrenia, including two sets found also in the CNV analysis, encoding proteins of the voltage-gated calcium channel and 5-HT2C receptor complexes. These results confirm studies on rare and common variants involving calcium channel genes in schizophrenia [145]. There is a convergence of data between genes containing rare variants of schizophrenia and those of GWAS in favor of two major functions: glutamatergic synaptic neurotransmission and calcium channels.

Several SNPs and CNVs located in genes associated with glutathione metabolism have been associated with schizophrenia. Glutathione plays an important role in brain protection against oxidative stress as an intracellular antioxidant or regulator of the redox system. Oxidative stress induced by a redox imbalance may particularly play a role in schizophrenia. Neurons are vulnerable to damage by oxidative stress and the presence of oxidative stress is found in many tissues of patients with schizophrenia. Glutathione can bind to the redox site on the NMDA glutamatergic receptor. A rise of extracellular glutathione levels increased glutamate-induced neuronal depolarization via NMDA receptor activation. Studies of genetically modified mouse models with low glutathione levels show many similarities to schizophrenia, including NMDA receptor hypofunction, alteration of inhibitory neurons to parvalbumin and impaired cognitive behavior. In addition, these mice have myelinization defects of many cortical bundles, as observed in patients, suggesting impaired connections by redox deregulation during development. Glutathione levels appear to be lower in individuals with schizophrenia in the medial prefrontal cortex and higher in the medial temporal lobe than in healthy controls [147].

Table 3 and Table 4 summarize findings related to genomics and schizophrenia. Interestingly, many CNVs (deletion/duplication) associated with schizophrenia are observed in autism spectrum disorder but also in intellectual disability, developmental delay, epilepsy, learning disorder, bipolar disorder, and many other neurodevelopmental disorders (see Table 4), suggesting the existence of common genetic vulnerability shared by these disorders. However, when Kirov et al. [133] scanned the genome for CNVs involved in schizophrenia, but also in autism spectrum disorder, developmental delay, and intellectual disability, they compared the risk for carriers of these variants to develop one or more of these disorders, i.e., their genetic penetrance, and showed that penetrance for schizophrenia was several times lower than for the other disorders. In any cases, the fact that many CNVs (deletion/duplication) found in schizophrenia are also observed in other psychiatric/mental disorders, strengthen the need to develop a dimensional and transnosographic approach in research on schizophrenia.

Furthermore, it is noteworthy that some CNVs implicated in schizophrenia contain no protein-coding genes, such as moderate repeats of DNA sequence (ribosomal DNA: rDNA) coding for ribosomal RNA, with only a fraction of them transcriptionally active [150]. rDNA copy number is elevated in the genomes of individuals with schizophrenia [151] and could explain the negative comorbidity reported between schizophrenia and rheumatoid arthritis [150,152,153]. Ribosomal repeats are hypothesized to play a role in the pathogenesis of schizophrenia and autism spectrum disorder (ASD). For example, a carrier of very low copy number of ribosomal genes is expected to have a milder form of ASD than an individual with the same epigenetic and genetic background, but with a higher ribosomal gene dosage [154]. Another non-coding repeats observed in schizophrenia are copy number variations of satellite III (1q12). Cell content of the Sat III repeat in leukocytes of patients with schizophrenia is significantly reduced compared to healthy controls. It has been suggested that the low amount of Sat III DNA is one of the possible causes of oxidative stress in the body of patients with schizophrenia [155]. The involvement of non-canonical regions of the genome may explain the genetic predisposition observed in schizophrenia as shown by twin studies, and the absence of strongly associated causative genes in most cases. Future studies are required to understand better the genetic contribution to schizophrenia of repeats of no protein-coding genes.

Taken together, genetic studies in schizophrenia show that many genetic variants have been identified, but none with high penetrance. The genetic contribution in schizophrenia is complex, not responding to a monogenic or oligogenic model. It is therefore likely the combination of multiple genetic variants at risk with a modest effect in an individual that leads to genetic vulnerability beyond a certain threshold. In this case, we speak of multifactorial and polygenic disease. The highly polygenic nature was demonstrated in 2009 by estimating a risk score involving thousands of common variants with very low effects on schizophrenia [156,157]. A genetic predisposition with the existence of mutations or genetic variants is not enough to develop the disease which will be expressed under the influence of environmental factors. Environmental factors have effects on genetic factors through epigenetic modifications which modulate the expression of certain genes (histone modification, chromatin remodeling, and DNA methylation). Very early gene/environment interactions involve some genes of neurodevelopment. Late gene/environment interactions involve genes responsible for dysfunction of the dopaminergic/glutamatergic system or synaptic plasticity. These genetic abnormalities could increase neuronal vulnerability to traumatic or toxic factors. Toxic factors such as regular cannabis intake, especially for early onset consumption or high cumulative dose of THC (tetrahydrocannabinol), could interact with genetic vulnerability factors to contribute to the development of psychosis. Childhood and adolescence are critical periods of vulnerability to environmental impacts, evoking the importance of physical and psychological trauma in the history of psychotic patients. Several models of stress vulnerability in schizophrenia have been proposed and abnormal stress responses of the hypothalamic–pituitary–adrenal axis have been reported in schizophrenia, as in autism spectrum disorder which shares certain common genetic and physiological pathways with schizophrenia, especially with EOS [20,158,159]. Animal models are of great interest to study Gene X Environment interactions in schizophrenia even if these animal models offer more a dimensional approach rather than providing specific categorical models of schizophrenia [160,161]. The Gene X Environment interaction model shows that additional stress in young animals leads to a permanent deficit in parvalbumin neurons and their perineuronal protection against oxidative stress, whereas it remains without ant effects in adult animals [162]. Furthermore, Janssen et al. [163] established a strong dose–response relationship between childhood abuse and psychosis after tracking 4045 people in the general population for two years. These data support a significant role of genetic factors and the implication of environmental stressful events in the development of the disease, suggesting that schizophrenia is a neurodevelopmental disorder.

Given the complexity of the genetics of schizophrenia, genomic data are insufficient to obtain a functional vision of the system; it becomes essential to be able to combine these data with those obtained by functional genomic techniques which explore the expression of genes in level of transcriptome, proteome, and metabolome. These data will open a new field of understanding of the various molecular mechanisms that influence the development of schizophrenia and the various symptomatology that result from it. The candidate genes approach is still relevant to understand these molecular mechanisms. It makes it possible to analyze the various potential targets with a more integrative and global approach using specific animal or cellular models for these susceptibility genes. In line with this strategy, genetically modified mice have been developed to determine better the role of different NMDA receptors in cognitive functions. These studies made it possible to explore individually the influence of the different specific subtypes of NMDA receptors [111].

## 5. Minor Physical Anomalies

In 1985, Kraepelin reported morphological abnormalities in patients with *dementia praecox* [164]. More recent studies agree that some morphological abnormalities are more common in individuals with schizophrenia compared to the general population—including palatal arches, epicanthus and telecanthus, bradycephaly, and prominent ears [165]. The prevalence of minor physical abnormalities could reach 62.7% according to some studies in patients with first-episode psychosis but with a great variety in the observed abnormalities [166]. The variety of minor morphological abnormalities is described in a meta-analysis of 13 studies [167]. These abnormalities are not specific to schizophrenia but could help in identifying earlier this psychiatric disorder, especially if they are associated with genetic and/or environmental risk factors for schizophrenia (including family history of schizophrenia). However, three signs appear to be robust: facial asymmetry, anomaly of the palatal vault, and atypical capillary implantation [167]. A prospective study [168] showed that the number of minor morphological abnormalities measured in children between 11 and 13 years was a risk factor for the onset of schizophrenia in adulthood (RR = 3.5). These anomalies were also associated with age at onset of schizophrenia and not with clinical severity; more precisely, minor physical anomalies were more frequent in patients with early onset schizophrenia (i.e., with onset before age 18 years) compared to later onset patients [169]. Missaoui et al. [170] confirm these previous studies. They reported that EOS patients, besides showing a greater severity of schizophrenia and negative symptomatology compared to individuals with later onset schizophrenia, had also more frequently minor physical and neurological abnormalities, including abnormalities of the face, hands, and ears. Gourion et al. [164] showed also that patients with schizophrenia and their parents have minor morphological abnormalities more frequently than non-affected individuals. The interest of these results is to highlight the possible role of genetic and/or environmental prenatal risk factors for schizophrenia (such as maternal infections or maternal bleeding, exposure to toxins, fetal anoxia [171]). Minor physical abnormalities would be the clinical expression of disturbances in the early development of the central nervous system (including the development of the ectoderm and neural ridges) and would therefore be in favor of the neurodevelopmental hypothesis [168]. 

Already, in 1997, Lewis reported a global reduction from 5% to 8% of cortical grey matter in patients with schizophrenia, not correlated with the duration of schizophrenia, suggesting the existence of an early neurodevelopmental process rather than a degenerative one [172]. More recently, Shenton and Kubicki [173] concluded that neuroimaging abnormalities found in individuals with schizophrenia are in favor of connectivity problems between brain areas, probably stemming from neurodevelopmental impairments. Several brain morphological changes have been described in schizophrenia based on neuroimaging which can reflect the impact of genetic but also environmental factors on brain structure and function [174,175]. More precisely, decreased cerebral volume (post-mortem examinations and in vivo neuroimaging) have been reported in schizophrenia. Volume changes mainly affect the frontal, prefrontal, and temporal regions, as well as limbic structures. Imbalance between grey and white matter, consisting of excessive grey matter loss and reduced white matter integrity, was also reported (for a review, see Wilczynska et al. [174]). Microscopic studies observed changes in neuronal density, myelin structure and astroglia and oligodendroglia, as well as size and shape of dendritic spines within synaptic connections. These findings are consistent with studies on activity of immunohistochemical markers such as parvalbumin, microtubule associated proteins or receptor markers, as well as neurofunctional imaging where impairments in blood flow, glucose metabolism and phospholipid catabolism are found within the atrophic structures. Some of those changes are related to clinical characteristics or factors such as severity of symptoms, type of applied treatment or neurological soft signs (these clinical signs were found to be associated with cortical, thalamic, and cerebellar changes in schizophrenia [174,175]). These brain morphological changes, taken together, support the neurodevelopmental hypothesis. In this context, research on minor morphological abnormalities as developmental markers of vulnerability highlights the importance of clinical genetic examination to detect dysmorphic features, rarely explored systematically in patients with psychiatric disorders but very useful for identifying genetic disorders associated with schizophrenia (see Table 2) and guiding diagnostic strategy (neuroimaging, oriented by the clinical results from clinical genetic and neurological examinations, can be requested if necessary).

## 6. Conclusions: Importance of a Clinical Genetic Examination in Schizophrenia

In the light of our observations, a clinical genetic examination appears to be an essential prerequisite for UHR individuals, as it allows an extended physical examination of the whole body to detect morphological abnormalities, including minor physical anomalies. It could be conducted on any patients showing prodroma signs of schizophrenia, searching for neurodevelopmental abnormalities in favor, as seen in this article, of vulnerability factors to schizophrenia. This examination should be performed by a trained clinical geneticist to detect dysmorphic features or malformations. It includes photos of the head (face and profile) with a good visibility of eyes and ears, as well as hands (palm and dorsal view) and feet. In the absence of easily access to clinical geneticists, the alternative option of using Face2gene software (developed by FDNA: Facial Dysmorphology Novel Analysis) is possible as a first step of exploration before a clinical genetic examination [176]. This software is a fast and accessible tool allowing the detection of dysmorphic signs based on the computer analyses of facial pictures with a reliable reproducibility. Its use in current practice allows an exploration of morphological abnormalities with clinical acceptability, high feasibility, and privileged access to the developmental history of patients. Finally, a physical examination of clinical genetics has an important role in the strategy of early identification of individuals with schizophrenia and genetic disorders associated with schizophrenia. Clinical genetic evaluation can greatly benefit from examining children and adolescents repeatedly over time to follow their developmental trajectory, including the evolution of morphological anomalies, and from examining at the same time the patient, his or her parents and siblings to compare the findings. The identification of genetic disorders associated with schizophrenia based on clinical genetic examination, has practical implications for diagnostic strategies, early detection or prevention of co-morbidity, specific treatment, follow up, and genetic counseling.

## Figures and Tables

**Table 1:** Concordance rates for schizophrenia in monozygotic twins and dizygotic twins.

	Monozygotic		Dizygotic	
Authors	Total Number of Twin Pairs	Pairwise Concordance Rate (%)	Total Number of Twin Pairs	Pairwise Concordance Rate (%)
Luxenberger, 1928 [48]	19	58	13	0
Rosanoff et al., 1934 [49]	41	61	53	13
Kallman, 1953 [50]	174	69	296	11
Slater and Shields, 1953 [51]	37	65	58	14
Inouye, 1963 [52]	58	59	20	15
Tienari, 1963 [53]	17	35	20	13
Kringlen, 1967 [54]	55	45	90	15
Fischer et al., 1969 [55]	25	36	45	17
Essen-Möller, 1970 [56]	11	64	27	15
Gottesman and Shields, 1972 [39]	22	58	33	12
Fischer, 1973 [57]	21	56	41	27
Kendler and Robinette, 1983 [58]	164	31	268	7
Onstad et al., 1991 [59]	24	48	28	4
Franzek and Beckmann, 1998 [60]	9	67	12	17

**Table 2:** Main genetic disorders associated with symptoms of schizophrenia and other psychiatric disorders.

Genetic Disorder	Estimated Rate of the Disorder in SCH (%)	Estimated Rate of SCH in the Disorder (%)	Degree of Intellectual Disability (ID)	SCH onset (VEOS, EOS, AOS)	Other Disorders	Morphological Abnormalities
NPC1 18q11.2 (Niemann-Pick type C disease) [68,69,70]	NA	5	Normal	AOS	-Depression -OCD -Bipolar disorder -Learning disability -Loss of intellectual ability	NA
22q11.2 deletion (DiGeorge/Velocardiofacial syndrome) [71,72]	2	30	Normal to severe ID	EOS to AOS	-Anxiety -OCD -ADHD -ASD	Long and narrow face, narrow palpebral slits, flat cheeks, prominent nose, small ears, small mouth, retracted chin
22q13 duplication SHANK 3 (Phelan-McDermid syndrome) [73,74]	NA	NA	Normal to severe ID	VEOS to AOS	-ASD -ADHD	Elongated head, high forehead, ptosis, long eyelashes, flat nose with a wide base, large ears
Xq13 (Lujan-Fryns syndrome) [75,76]	NA	NA	mild to moderate ID	EOS to AOS	ASD	Long and narrow face, maxillary hypoplasia, small mandible, and prominent forehead
15q21.1 Fibrillin mutation (FBN1) (Marfan syndrome) [77,78]	NA	>1	Normal	AOS	-Bipolar disorder -Depression - ADHD	Large size, arachnodactyly, pectus excavatum, pectus carinatum, micrognathia, ogival palate, tooth overlap, scoliosis, overlaxity
Angelman syndrome (maternal 15q11-q13 deletion, paternal uniparental disomy, mutations of *UBE3A* that encodes an ubiquitin E3 ligase [79,80] Prader-Willi syndrome (maternal uniparental disomy at 15q11-q13, paternal deletions) [79,81]	7.9	NA	Mild to moderate	NA	-Developmental delay -ASD -ID -OCD	Obesity, growth delay and hypogonadism, facial dysmorphism, hypotonia
Reciprocal 7q11.23 duplication syndrome [82,83,84,85]	6.6	NA	Mild to moderate	NA	-ASD -ID -Anxiety	Facial dysmorphism, short stature, heart and endocrine malformations, hypercalcemia
HBMS 11q23.3 (acute intermittent porphyria) [69]	NA	4	Normal	AOS	-Depression -Anxiety	NA
del/dup 1q (Homocystinuria) [86,87]	NA	NA	Normal to severe ID	EOS to AOS	-OCD -Depression -Anxiety	Genu valgum, hollow foot, dolichostenomelia, pectus excavatum or carinatum, kyphosis, scoliosis, long face, high forehead, large, floppy, and low-set ears, and flat philtrum
ARS-A 22q (metachromatic leukodystrophy adult) [88]	NA	50	Normal to severe ID	EOS to AOS	-Ataxia -Loss of intellectual ability	NA
13q14.3 (Wilson’s disease) [89]	NA	1,4 à 11,3	Normal to moderate ID	NA	-Depression -Anorexia	NA
PDE4B 1p31 [90]	NA	NA	Normal to mild ID	NA	-Bipolar disorder -Anxiety	NA
DISC1 t(1;11)(q42;q14) [91,92]	NA	NA	Normal to mild ID	AOS	-Bipolar disorder -Anxiety	NA
1q21.1 Copy-Number Variation (CNV) (1q21.1 del/dup) [93,94,95]	15	NA	Normal to mild ID	NA	-Developmental delay -Learning disability -Anxiety -ADHD -ASD	Microcephaly (deletion) macrocephaly (duplication)
15q13.3 CNV (15q13.3 del) [96,97,98]	15	NA	Mild to moderate ID	NA	-Developmental delay -Learning disability -ADHD -ASD	Hypertelorism, synophrys, prominent philtrum, everted upper lip, hypotonic facies, upslanting palpebral fissures, brachycephaly
Xp22 CNV (Xp22 del) [99]	NA	NA	Normal to mild ID	VEOS to AOS	Learning disability	Low-set ears, anteverted nostrils, small size
16p11.2 CNV (16p11.2 dup) [100]	NA	NA	Normal to mild ID	VEOS	-ASD -Bipolar disorder	Macrocephaly

Note: SCH = Schizophrenia, NA = Not Available, VEOS = Very Early Onset Schizophrenia, EOS = Early Onset Schizophrenia, AOS = Adult-Onset Schizophrenia, OCD = Obsessive Compulsive Disorder, ASD = Autism Spectrum Disorder, ADHD = Attention Deficit Hyperactivity Disorder, ID = Intellectual Disability.

**Table 3:** Candidate genes and loci associated with schizophrenia in cytogenetic, linkage, and genome wide association studies.

Genomic Region and Candidate Loci	Gene	Protein
Vulnerability genes for schizophrenia based on cytogenetic studies		
t (1 ; 11) (q42.1; q14.3)	*DISC1*	Disrupted in schizophrenia 1 or DISC1
22q11.21 Deletion	*COMT*	Catechol O-methyltransferase
Candidate genes for schizophrenia supported by linkage findings		
1q23.3	*NOS1AP* *RGS4*	Carboxyl-terminal PDZ-bonding domain of neuronal nitric oxide synthase 1 adaptor protein Regulator of G-protein signaling 4
6p22.3	*DTNBP1*	Dystrobrev in binding protein 1
8p12	*NRG1*	Neuregulin 1
13q33.2; 13q34	*DAOA*	D-amino acid oxidase activator
Single Nucleotide Polymorphism (SNP) supported by genome wide association studies (GWAS)		
11q23.2	*DRD2*	Dopamine receptor D2
7q21.11-q21.12	*GRM3*	Glutamate metabotropic receptor 3
16p13.2	*GRIN2A*	Glutamate ionotropic receptor NMDA type subunit 2A
17p13.3	*SRR*	Serine Racemase
5q33.2	*GRIA1*	Glutamate ionotropic receptor AMPA type subunit 1
12p13.33	*CACNA1C*	Calcium voltage-gated channel subunit alpha 1 C
10p12.33-p12.31	*CACNB2*	Calcium voltage-gated channel auxiliary subunit beta 2
22q13.1	*CACNA1l*	Calcium voltage-gated channel subunit alpha 1 l

**Table 4:** Copy number variants (CNV) associated with schizophrenia (SCZ) and other disorders.

Region and CNV Type (Microarray)	Candidate Genes in the Region	Phenotypes *
1q21.1 Deletion/Duplication	*HYDIN*	SCZ, ASD, ID, DD, ADHD, Anxiety, LD, IGE deficit
2p16.3 Deletion/Duplication	*NRXN1*	SCZ, ASD, ID, TS
3q29 Deletion/Duplication	*PAK2*, *DLG1*, *PAK3*, *DLG3*	SCZ, ASD, ID, ADHD, EP
7q11.23 Duplication (Williams–Beuren syndrome region)		SCZ, ASD, ID, EP
7q35-q36 Deletion	*EN2*, *CNTNAP2*	SCZ, ASD, ID, EP
15q11.2 Deletion (Angelman syndrome/ Prader-Willi syndrome region)	*CYF1P1*, *TUBGCP5*, *NIPA1*, *NIPA2*	SCZ, ASD, ID, DD, OCD, IGE deficit
15q11-13 Duplication	*GABRA5*, *GABRB3*, *GABG3*, *UBE3A*, *SNRPN*, *CHRNA7*	SCZ, ASD, ID, EP, Ataxia
15q13.3 Deletion/Duplication	*CHRNA7*, *OTUD7A*	SCZ, ASD, ID, DD, ADHD, LD, EP
16p11.2 Deletion/Duplication	*DOC2A*, *ERK1*	SCZ, ID, DD, BD, EP, LD
16p13.11 Deletion/Duplication	*NDE1*	SCZ, ASD, ID, ADHD, EP, IGE deficit
17q12 Deletion/Duplication	*Undefined*	SCZ, ASD, ID, EP
22q11.2 Deletion/Duplication (DiGeorge/Vélocardio-facial syndrome region)	*PRODH*, *COMT*, *DGCR6*, *TBX1*, *CRKL*, *FGF8*	ASD, SCZ, ID, DD, ADHD, EP, LD
22q13.3 Deletion	*SHANK3*	SCZ, ASD, ID, DD
Xp22.31 Deletion/Duplication	*NLGN4*	SCZ, ASD, ID, BD, TS
Xq13.1 Deletion/Duplication	*NLGN3*	SCZ, ASD, ID, BD

Adapted from Tordjman et al. [20], Vassos et al. [72], Lacy and King [148], Malhotra and Sebat [149]. * Note: SCZ, Schizophrenia; ASD, Autism Spectrum Disorder; ID, Intellectual Disability; ADHD, Attention Deficit Hyperactivity Disorder, DD, Developmental Delay; BD, Bipolar Disorder; TS, Tourette syndrome; OCD, Obsessive Compulsive Disorder; EP, Epilepsy; LD, Learning Disorder.
